# The Value of Pain Coping Constructs in Subcategorising Back Pain Patients according to Risk of Poor Outcome

**DOI:** 10.1155/2013/898573

**Published:** 2013-10-24

**Authors:** Nicholas Harland, Cormac Gerard Ryan

**Affiliations:** ^1^The School of Health and Social Care, Teesside University, Tees Valley, Middlesbrough TS1 3BA, UK; ^2^South Tees Hospital Foundation Trust, Marton Rd, Middlesbrough TS4 3BW, UK

## Abstract

*Background.* Subcategorising patients with chronic low back pain (CLBP) could improve patient outcomes and facilitate prioritisation of treatment resources. *Objective.* This study aimed to develop a subcategorising method for individuals with CLBP using the Coping Strategies Questionnaire 24 (CSQ24) and to investigate the methods potential validity. *Methods.* 196 patients were recruited from a physiotherapy outpatients department. All participants completed a battery of questionnaires before and after treatment including the CSQ24 and a measure of pain, disability, and mood. At discharge participants also completed a global subjective outcomes scale consisting of a 6-point Likert scale. All participants received usual physiotherapy. *Results.* Cut-off values for the CSQ24 were calculated using triangulation of the findings from three different statistical methods. Cut-off values were identified for the Catastrophising and Cognitive Coping subscales of the CSQ24. Participants were categorised into low, medium, and high risk of a poor outcome. The cut-off values for these were ≥21 on Cognitive Coping and ≤9 on Catastrophising for low risk and ≤15 on Cognitive Coping for high risk, with all other patients being classified as being at moderate risk. *Conclusion.* Further validation is required before this approach can be recommended for clinical practice.

## 1. Introduction

Back pain constitutes a significant health problem, with a lifetime prevalence of 80–85% [1]. Despite this, our understanding of the condition remains relatively poor and it has been said that “For one of the most common and debilitating conditions we have no real answers” [2]. Exercise based therapy combined with adjunctive treatment such as manipulations or acupuncture delivered within a psychosocially aware environment can be beneficial for many patients [3, 4]. However, a significant number of patients continue to have variable longer term outcomes from treatment and between 42% and 75% continue to suffer some degree of back pain a year after its first onset [3].

One potential theory for the lack of consistent treatment success may be attributable to the fact that patients with nonspecific low back pain (LBP) have been considered within the literature as a homogenous group [5]. It is hypothesised however that within the broad diagnostic category of nonspecific mechanical low back pain there are a number of subgroups which could respond well to specifically tailored interventions. 

This theory is dependent on the idea that LBP patients can be subcategorised into more homogenous subgroups and, following this, treatments that best suit each group can be developed and tested. There is growing interest into which constructs or sets of constructs might be most appropriate to categorise patients into subgroups [6–8]. One of the earliest subgrouping methods for LBP, the McKenzie method, was proposed by McKenzie and May in 2003 [9]. The McKenzie method subgrouped patients into three broad categories: postural syndrome, dysfunctional syndrome, or derangement syndrome, based upon their pain response to movements in different directions during assessment [9]. Though still commonly used in clinical practice, there is limited evidence for the effectiveness of this classification system [10].

A more recent movement-based subgrouping system proposes that maladaptive motor behaviour exposes the spine to on-going strain resulting in pain [11]. The system combines a patho-/anatomical and psychosocial approach for diagnosis and management. The system proposes five directional subgroups of which flexion pattern and active extension pattern are the most common ones [11]. While there is growing evidence around the validity and reliability of this approach for sub classification [12–14], there remains little evidence of its clinical effectiveness in prospective intervention studies.

Over the last five years the STarT back screening tool (SBT) has shown promising results as a potential subgrouping tool [15, 16]. The SBT uses multidimensional variables including pain, disability, and psychosocial constructs to categorise patients as low, medium, or high risk of a poor outcome [15]. Results from a recent randomised controlled trial, which used this subgrouping protocol to inform a subsequent pathway of care, have been promising. Patients who received stratified care informed by the STarT back protocol had improved and had more cost effective outcomes at 12 months [16].

A number of predominantly psychosocial variables [6–8, 11, 17, 18] have been shown to have a relationship with poor outcome and/or work loss in chronic back pain. The majority of such studies however exclude analysis or findings that allow useful application of results to clinical practice. This is because such studies often fail on two issues: (1) to identify and validate subgrouping cut-off points within the tools examined and (2) to test the clinical benefit of targeting tailored treatment to these subgroups in prospective RCTs. 

The STarT program [16] is one of the few instances where these two issues have been addressed. The issue remains however that many studies, including STarT back, use mixed sets of constructs, such as pain, disability, and a range of psychosocial or quality of life variables to subgroup patients with LBP. While using a mixture of variables has shown some efficacy, it does not help identify which constructs are most useful for subcategorisation purposes. As a result it is possible some variables used in categorisation tools may be redundant. Identifying and examining a specific construct, such as coping, and establishing its specific subcategorisation properties would help start addressing this issue.

Investigation to discover the relative value of specific constructs like pain, disability or coping to subcategorise patients may also facilitate the development of more specifically tailored treatment programs to address the particular issues associated with that construct. If specific constructs within coping linked to poor outcome could be identified, for instance, then a treatment approach to address that specific construct could be designed and prospectively tested. Multidimensional categorisation tools, though having some efficacy, do not facilitate the development and testing of novel treatment approaches. If the value of a single construct, such as coping, in categorising patients can be identified, then novel approaches aimed specifically at addressing issues related to the fact that construct could be tested. 

The aim of this study was therefore to (1) develop a high, medium, and low risk based subcategorising protocol for patients with LBP using the constructs measured by the Coping Strategies Questionnaire 24 (CSQ24) [19] and (2) investigate the potential validity of the protocol developed to inform future study.

The CSQ24 [19] was chosen as the tool to be evaluated as it is recommended for use [20] over the original CSQ [21] which remains one of the longest standing and frequently researched coping tools internationally [22]. As clinical use of categorisation methods and subsequently stratified care also require repeated testing, the tool used also needs to prove longitudinal validity and this has recently been established for the CSQ24 [23]. It is also often noted that fear, as measured by Catastrophising within the CSQ and CSQ24, explains a greater proportion of variance in disability in back pain than do many other constructs [24]. Further than this, however, conceptually the CSQ24 has an advantage over other commonly used tools in the field, like the fear avoidance beliefs questionnaire (FABQ) [25], as, in addition to measuring the presence of negative constructs such as fear, it is the only tool of its kind that measures a positive construct, in this case Cognitive Coping. As the absence of a negative construct does not necessarily mean the presence of a positive construct and when identification of patients at the lowest risk of poor outcome is a goal, a tool identifying both negative and positive constructs is at least conceptually advantageous.

## 2. Methods

### 2.1. Inclusion Criteria

Patients were recruited from a sample completing physiotherapy treatment after referral from a back pain assessment clinic. Participants were included if they were aged 16–85 years and had chronic low back pain [CLBP] (with or without leg pain) for ≥3 months of duration. Individuals were excluded from the study if they had any red flags indicating serious pathology, if they had any medical condition that would contraindicate physiotherapy treatment or if they lacked the capacity to give informed consent.

### 2.2. Procedure

Participants recruited into the study completed a battery of questionnaires before and after physiotherapy treatment. The battery of questionnaires included the Coping Strategies Questionnaire 24 (CSQ24) [19], a pain visual analogue scale (VAS) [26], The Roland and Morris disability questionnaire 18 (RMDQ-18) [27], and the Hospital Anxiety and Depression (HAD) scale [28]. At discharge patients also completed a global subjective outcomes scale consisting of a 6-point Likert scale with response points labelled from “I Feel Worse” to “Completely Better.” A range of participant demographic information was also recorded including age, gender, duration of symptoms, and comorbidities. Participants received usual care physiotherapy. The number and type of treatments provided to each participant were recorded in addition to total length of time under treatment.

### 2.3. Materials

Participant coping was assessed using the CSQ24. The CSQ24 [19] is a shortened version of the Coping Strategies Questionnaire (CSQ) [20] recommended for use over the original version [28]. The CSQ24 consists of 23 items about coping, with a 7-point Likert scale anchored with “Never Do That” and “Always Do That,” and one item measuring perceived control over pain with a 7-point Likert scale is anchored with “No Control” and “Complete Control.” The CSQ24 has four subscales: Catastrophising, Diverting Attention, Reinterpreting, and Cognitive Coping; each subscale is scored from 0 to 36. The CSQ24 has demonstrated a good level of validity as a measure of coping in individuals with CLBP [19, 23]. 

Pain was assessed using a VAS anchored with “No pain” and “Worst Possible Pain” and referring to “average pain over the last week.” The VAS is a valid and reliable measure of pain [25]. Function was measured using the RM-18, a commonly used back pain specific questionnaire consisting of 18 “yes” or “no” items. A higher score suggests poorer levels of functioning. The questionnaire has demonstrated good levels of validity as a measure of physical function in patients with CLBP [27].

The Hospital Anxiety and Depression (HAD) scale has been used to assess participants' levels of anxiety and depression. The scale consists of 14 items measured on a 4-point Likert scale with varying anchoring statements. The scale is scored from 0 to 21 for both anxiety and depression with lower scores indicating less presence of the construct. The scale has been noted to be valid and reliable for individuals with CLBP [28]. 

At discharge, participants completed a global subjective outcomes (GSO) scale consisting of a 6-point Likert scale with response points labelled from “I Feel Worse” to “Completely Better.” Such scales are valid for use in clinical settings [29] and are seen as the “gold standard” for use when establishing clinically significant change, and subsequently they represent a highly relevant anchor variable when linking clinical assessment scores to outcomes in subcategorisation studies [30, 31].

### 2.4. Analysis

Establishing multiple valid cut-off points using a psychosocial variable to establish risk of poor outcome is difficult. No gold standard protocol exists, and frequently employed methods of establishing a single cut-off point such as using receiver operating characteristic (ROC) curve analysis only output a binary categorisation so cannot be used. As an alternative to ROC curve analysis in the establishment of clinically important change, indices using mean scores anchored to establish outcome indicators such as global subjective outcome can be used. This method has been employed in articles used to establish an international consensus regarding pain measurement [32]. This method also seems valid in this case where multiple screening cut off points are needed. 

To maximise validity a triangulation approach using three different but complementary methods was chosen to establish the cut-off point for patients at high, medium, and low risk of poor outcome using the CSQ24. The findings from these three methods were compared to identify the most appropriate cut-off values to subcategorise the participants. Only the Catastrophising and Cognitive Coping subscales were used to establish cut-off scores, as a large sample study suggests that most respondents tend to score highly on either Cognitive Coping or Catastrophising [33], with few respondents scoring highly on more than one subscale concurrently. Evidence also suggests that Reinterpreting may have poor construct validity and Diversion scores remain difficult to be interpreted regarding their positive or negative effect on the individual [19].

#### 2.4.1. Method One

The first method was based upon the suggestion by Harland and Georgieff [19] that, as the majority of respondents will have some degree of positive score on each of the CSQ24's four constructs, the construct attracting the highest score should be seen as that individuals' dominant coping strategy. With Catastrophising being seen as a negative marker of outcome [34–36] and Cognitive Coping being seen as a positive marker [37–39] the average score for Catastrophising and Cognitive Coping was calculated after examining the cases at baseline assessment, according to dominant coping strategy (Cognitive Coping or Catastrophising) at the time. 

#### 2.4.2. Method Two

The second method calculated the average assessment score for both Cognitive Coping and Catastrophising for those patients that, at discharge, were either “a lot better” or “completely better” using the GSO scale. This would provide figures that could be used to identify those patients at the least risk of poor outcome. When definitive identification of cases at the least risk of poor outcome was needed and when evidence shows that many patients who successfully complete treatment continue to have pain to some degree for a year or more afterwards [3], it was not felt valid to include the “moderately better” outcomes in this method.

#### 2.4.3. Method Three

The third method used HAD scores as an anchor to relate to mean Catastrophising and Cognitive Coping scores as anxiety and depression have significant correlations with multiple other back pain measures [40–44]. The data was split according to an established cut-off point of 12 or more for anxiety or depression, this cut off identifying patients likely to be negatively affected and therefore potentially more difficult to treat [45]. Data from the study was examined and the average Catastrophising and Cognitive Coping score was calculated for participants according to whether they scored 12 or more on either anxiety or depression using the HAD.

## 3. Results

### 3.1. Participant Characteristics

196 cases were recruited into the study. The average age of participants was 52 years (SD 14.59) and 42% (*n* = 81) of participants were male. 13% (*n* = 26) of participants were not working due to their back pain and 41% (*n* = 80) had had back pain for over 10 years. The treatment received by participants was predominantly exercise based (89% of cases) and patients received between 2–4 treatments over a period of ~11 weeks. Treatment was not controlled to ensure that results remain relevant to normal clinical situations.

### 3.2. Method 1

When the average Catastrophising and Cognitive Coping score is calculated regarding individuals who scored most highly on the Catastrophising scale at assessment, theoretically therefore being at greater risk of poor outcome (12.8% of participants, *n* = 24), the scores are 23.29 and 12.79, respectively. Individuals scoring more highly than 23 on Catastrophising and less than 13 on Cognitive Coping at assessment could therefore potentially be seen as being at higher risk of poor outcome. 

When the average Catastrophising and Cognitive Coping score is calculated regarding individuals who scored most highly on the Cognitive Coping scale at assessment, theoretically therefore being at lower risk of poor outcome (65.2% of participants *n* = 124), the scores are 7.16 and 23.66, respectively. In this case a score of 7 or less on Catastrophising and of 24 or more on Cognitive Coping could be used. Pragmatically these cut-off scores have little value when the more simple method of using the dominant coping strategy at the time can be used, but, in this case, when triangulation around proposed scores is being attempted, they represent a theoretically sound base line.

### 3.3. Method 2

The average scores for those shown to be at low risk of poor outcome, reporting to be “a lot better” (*n* = 121, 61.7%) or “completely better” (*n* = 9, 4.6%) at discharge were 20.37 for Cognitive Coping and 9.84 for Catastrophising. The method of using global subjective outcome as an anchor to link coping scores was of limited use in identifying potential cut-off scores for patients at high risk of poor outcome. This was due to no patients reporting to be worse and only (*n* = 6, 3.1%) reporting to be the same at discharge from physiotherapy treatment.

This study recruited patients completing physiotherapy care rather than those unable to complete care and subsequently referred on for further opinion, such as from a surgeon. As a result, data was not gained from this group who may have shown unchanged or worsening clinical outcome scores. As outlined in the method section, it was not felt that scores from those reporting to be “moderately better” (*n* = 43, 21.9%) were definitive enough to be used in the production of a low risk of poor outcome category. The lack of data regarding unchanged or worsening patients is a limitation of this study.

### 3.4. Method 3

The cut-off values calculated for the CSQ24 using method 3 are shown in Table 1. For Catastrophising the mean difference between those scoring ≥12 or <12 was significantly different (*P* = .000) for both anxiety and depression, respectively, using an independent samples *t*-test. In the case of Cognitive Coping the mean difference between those scoring ≥12 or <12 for anxiety and depression was also significantly different (*P* = .004 and *P* = .000), respectively. This provides evidence of a statistically significant difference between these populations. The figures would suggest that a score of 20 or more on the Catastrophising scale, with this being a pragmatic number between the scores of 19.25 and 21.07 for anxiety and depression, respectively, could potentially be used as cut-off value for patients at high risk of poor outcome. A score of 15 or less on the Cognitive Coping scale could potentially be used for similar purposes.

Using the same strategy of examining mean scores of data split according to significant anxiety or depression, a cut-off score regarding Catastrophising and Cognitive Coping can also be established to identify patients potentially at least risk of poor outcome. In this case, as shown in Table 1, a score of 21 or more on Cognitive Coping or of 9 or less on the Catastrophising scale could potentially be used for this purpose.

### 3.5. Triangulation of Methods

The cut-off values for the three methods used are shown in Table 2. The three different analysis methods used to indicate possible CSQ24 cut-off scores produced broadly similar scores.

Using the results from method 3 that produced the median values for the different methods, figures of ≥21 on Cognitive Coping and ≤9 on Catastrophising could be used to identify low risk patients and figures of ≤15 on Cognitive Coping and ≥20 on Catastrophising could be used to identify high risk patients. The data was then examined to identify what proportion of cases would subsequently fall into each category if the protocol was applied to the study population.

If individuals had to meet both Catastrophising and Cognitive Coping cut-off scores to be categorised as high or low risk, 27% of patients would fall into the low risk category and 17% into the high risk category, leaving 56% in the moderate risk category.

## 4. Discussion

The aim of this study was to develop a method for subcategorising individuals with CLBP according to high, medium, or low risk of poor outcome using only the construct of coping as the subcategorising variable. In this way, in the developing field of subcategorisation and subsequently stratified care, the value of coping can be assessed and future studies are planned to test the protocol prospectively or compare figures with the outcome of other protocols.

Epidemiology figures suggest that a proportion of back pain patients are not improved significantly with treatment and/or continue to suffer disability in the long term [3]. It seems that, although patients may report improvement with physiotherapy or other care, that improvement may only be partial or temporary, and these cases continue to utilise health resources and report both pain and disability, as such, within this context, patients who may report a degree of improvement but present with risk factors such as high Catastrophising, anxiety, or depression may be at greater risk of on-going symptoms. The potential to identify these cases and tailor care accordingly to potentially improve the degree and longevity of outcome is appealing. 

Cut-off values have been derived from the Catastrophising and Cognitive Coping subscales of the CSQ24. This study provides evidence to suggest that back pain patients able to complete a course of physiotherapy care may be able to be categorised into low, medium, or high risk of a poorer overall outcome, using the scores suggested in Table 2. The three methods used to identify appropriate cut-off points for subcategorisation all produced broadly similar values adding confidence to the validity of the values identified. It is a point of strength of the study that a triangulation method was used successfully. In theory the cut-off values identified could be used to subcategorise patients for treatment purposes, though further validation is required before the categorisation process can be recommended for clinical practice.

According to the categorisation method used in this study 27% of patients were at low risk, 56% were at moderate risk and 17% were at high risk of a poor outcome. These findings are remarkably consistent with data from a study using the STarT tool applied to a similar physiotherapy patient population where 33% were categorised as low risk, 48% as medium risk, and 19% as high risk [46]. It is encouraging that the percentages found in this study are similar to those found by Fritz et al. [46], and this adds significant evidence of the potential validity of the values identified and provides some assurance that the limitations of the sample, only being those completing physiotherapy, have not led to significant skewing of the data gained.

Despite the similarities found with Fritz et al. [46] assessment of the percentage of patients that would fall into each category led to questioning whether both Catastrophising and Cognitive Coping criteria would need to be met in order to categorise patients. Certainly with knowledge of the relatively high number of CLBP patients who fail to improve significantly with treatment and/or have on-going pain, 17% did not seem to represent a realistic number of those at high risk. A further analysis was therefore performed to establish the percentage that would be gained if only the Catastrophising or Cognitive Coping cut-off points were used to categorise high risk patients. Under these terms, 13.5% and 25.5%, using the Catastrophising and Cognitive Coping cut-off points, respectively, would fall into a high risk group. 

When the nature of Cognitive Coping and Catastrophising is examined from a change perspective, Cognitive Coping has been shown to be resistant to change over a period of effective treatment, whereas Catastrophising has been shown to be variable in nature over time [23]. Taking these factors into account with Cognitive Coping apparently difficult to change with treatment and stable over time, therefore defining the patient more reliably than Catastrophising from a screening perspective, it is suggested that only Cognitive Coping is used to categorise patients into a high risk group. Both Catastrophising and Cognitive Coping score cut-offs could still be used to categorise into the low risk group.

The recommended protocol for categorising patients according to risk of poor outcome using the CSQ24 is shown as follows and in the study population would lead to 25.5% being high risk, 27% being at low risk, and 47.5% being at moderate risk of poor outcome.

 high risk: Cognitive Coping ≤15,
 low risk: Cognitive Coping ≥21 and Catastrophising ≤9,
 moderate risk: all cases not falling into high or low risk groups.

### 4.1. Limitations

A key limitation to the study is that no patients reported being worse and only six reported being the same at discharge from physiotherapy treatment. Patients receiving conservative management who worsened significantly would normally be referred on for alternative opinion and were not recruited into the study. The results of this study are therefore only applicable to a population of patients with manageable pathology who are able to complete a course of physiotherapy care. 

### 4.2. Conclusions

This study provides evidence to suggest that back pain patients able to complete a course of physiotherapy care may be able to be categorized into low, medium, or high risk of a poorer overall outcome. Although the cut-off values can be used to subcategorize patients for treatment purposes, further validations are required before the categorization process can be recommended as a standard for clinical practice.

## Figures and Tables

**Table 1:** Cut-off values for the Catastrophising and Cognitive Coping scores of the CSQ24 using method 3.

	Anxiety		Depression	
	≥12	<12	≥12	<12
	(*n* = 20)	(*n* = 168)	(*n* = 15)	(*n* = 173)
Coping (range 0–21)	16.25 (6.23)	21.48 (7.61)	14.40 (7.15)	21.49 (7.43)
Catastrophising (range 0–21)	19.25 (7.73)	9.33 (7.43)	21.07 (8.63)	9.46 (7.32)

Cut-off values are the mean scores for the Coping and Catastrophising subscale of the CSQ24 calculated from published cut-off values for the HAD scale. Data are presented as mean (1SD).

**Table 2:** Identified cut-off values for the CSQ24 using three different methods.

Risk	Catastrophising			Cognitive Coping		
	High	Medium	Low	High	Medium	Low
Method 1	≥23	8–22	≤7	≥13	14–23	≥24
Method 2	NA	NA	≤10	NA	NA	≥20
Method 3	≥20	10–19	≤9	≤15	16–20	≥21
Suggested scores	≥20	10–19	≤9	≤15	16–20	≥21

All scores are measured in a range from 0 to 36.
